# Editorial: Proteomics, mass spectrometry and bioinformatics in renal pathophysiology

**DOI:** 10.3389/fphys.2023.1338270

**Published:** 2023-11-23

**Authors:** Timothy D. Cummins, Visith Thongboonkerd

**Affiliations:** ^1^ Division of Nephrology and Hypertension, Department of Medicine, and Clinical Proteomics Center, University of Louisville, Louisville, KY, United States; ^2^ Medical Proteomics Unit, Research Department, Faculty of Medicine Siriraj Hospital, Mahidol University, Bangkok, Thailand

**Keywords:** bioinformatics, biomarker, mass spectrometry, proteomics, renal pathology, renal physiology

During the past 2 decades, proteomics, mass spectrometry and bioinformatics have been widely applied to study various kidney diseases, e.g., acute kidney injury (AKI), chronic kidney disease (CKD), diabetic kidney disease (DKD), and glomerular diseases. Such applications will surely lead to breakthroughs in mechanistic insights, biomarker targets for diagnostics/prognostics and best clinical practice. This Research Topic on proteomics, mass spectrometry and bioinformatics in renal pathophysiology offers a 5-paper selection analyses of a variety of kidney diseases using different Omics and bioinformatics approaches.

Zhu et al. provide in-depth plasma metabolomics analysis of 31 CKD patients without dialysis (or pre-dialysis) compared with 31 and 30 CKD patients who are on hemodialysis (HD) and peritoneal dialysis (PD), respectively. This approach provides important data on the plasma metabolome in CKD and its alterations by HD and PD. Insights into the disease mechanisms may be revealed using these detailed metabolomic findings to define the critical changes in metabolites (i.e., enzyme activity) that occur during disease progression and response to different modalities of the dialysis treatment. For example, metabolites related to cysteine metabolism predominantly differ between the pre-dialysis patients and those who are on HD or PD, whereas metabolites related to purine metabolism largely differ between the HD and PD groups. These results suggest useful metabolite candidates towards managing Stage 5 CKD patients with some insights into precision medicine, which may be more feasible by using more in-depth informatic analyses together with the targeted metabolite studies.

The Omics studies often generate large datasets with untapped information that can be gleaned with a deep-dive informatics modeling approach. Cai et al. provide a bioinformatics analysis identifying genes implicated in the immune response of membranous nephropathy (MN). Microarray datasets of 65 MN patients and 9 healthy individuals from the Gene Expression Omnibus database are mined for deeper insights. Using linear model for microarray data (LIMMA) and enrichment approaches, 370 differentially expressed genes in MN and 20 hub genes of their interacting network have been elucidated. Some of these bioinformatic data can be confirmed by experimental approaches (i.e., immunohistochemistry). These data clearly show that mining existing datasets is imperative towards improving our understanding and depth of the knowledge on the renal pathophysiology.

Implementation of the Omics approaches to improve decision making in clinical practice is a promise of precision medicine that begins to be more fully realized with better Omics utilization. Early precise decision making in the clinical toolkit will provide more targeted and efficacious treatments. Citrate dialysate has been shown to improve clinical outcome in HD patients, but the mechanisms of action are poorly understood. Broseta et al. provide a metabolomics analysis of plasma for improved understanding of utilizing citrate dialysate compared with acetate dialysate. Plasma metabolites are collected from pre, mid, immediately post and 30-min-post HD to provide a sequential profiling analysis and to determine differences from using different dialysates. Their analyses have revealed that citrate dialysate offers a better clearance of metabolites that belong to uremic toxins while increases the post-dialysis branched-chain amino acids. Moreover, citrate exclusively decreases metabolites related to lysine degradation, whereas the increased clearance of acylcarnitines is unique for acetate dialysate. These findings underscore that large-scale metabolome analysis can enhance our understanding of different therapeutic outcomes of dialysis using various kinds of dialysates as reflected by differential changes in the post-dialysis plasma metabolome.

Understanding the renal pathophysiology requires comprehensive analysis of all aspects of the kidney function at tissue, organ and cellular levels. Tools developed for analysis of transcriptomics, proteomics, metabolomics and lipidomics can often be cross-utilized for data mining and statistical interrogation. Development of single-cell analytical techniques allows for detailed mapping and delineation of origins of the disease pathophysiology. Mao et al. review the recent technological advances in single-cell transcriptomics and provide an overview of its application towards a better understanding of the pathophysiology of DKD specifically. Cell-type specific information in the context of changes in transcript level can give a precise molecular map or snapshot of the disease mechanisms. Integration of single-cell proteomics, metabolomics and transcriptomics can provide the most robust analysis, to date, and hence will be a major undertaking in a renal context to give a precision overview of cellular changes in DKD (and the renal pathophysiology in general).

Modeling of human diseases is a difficult but worthwhile undertaking when development of animal models in conjunction with the Omics methodology can be implemented to improve our understanding of complex kidney diseases. Exertional heat stroke (EHS)-induced AKI is prevalent in equatorial countries with large agriculture sectors, and biomarker efforts can benefit early and targeted treatment strategies. Understanding the onset of AKI with biomarkers can prevent or reduce effects of the EHS-induced AKI. Wen et al. pursue a proteomics analysis of the EHS-induced AKI in a rat model to determine candidate protein biomarkers of the disease onset. The analysis has revealed 3,129 differentially expressed proteins in EHS-induced AKI. Among these, 10 significantly modulated and disease-related targets can be verified in rat renal tissue and urine by quantitative RT-PCR and Western blotting. The initial analyses from model organisms and further translation into human diseases will be a critical step for implementing from pre-clinical target identification to bed-side clinical utility. The application and expansion of these approaches in diverse pre-clinical experiments will be important towards better and more precise utilization of proteomics technology in addressing gaps in our understanding of various kidney diseases.

Overall, the implementation of various Omics technologies to more comprehensively understand kidney biology and pathophysiology has taken great strides in the last 25 years. Basic research is now reaching the clinic and providing better decision-making tools and information to improve therapeutic outcome and patients’ quality of life and survival through critical stages of illness. Nevertheless, the promise of precision medicine is not fully implemented in the clinic and will take many more dedicated research efforts and translational applications to be better utilized for improving the therapeutic outcome. This Research Topic only highlights a few technical examples of the breadth of approaches and models for utilizing the Omics technologies in pre-clinical and clinical settings. Improvements in research methodology and bioinformatics mining approaches will undoubtedly lead to even more interesting and groundbreaking insights into the renal pathophysiology.

